# The Missing Heritability in T1D and Potential New Targets for Prevention

**DOI:** 10.1155/2013/737485

**Published:** 2013-03-13

**Authors:** Brian G. Pierce, Ryan Eberwine, Janelle A. Noble, Michael Habib, Hennady P. Shulha, Zhiping Weng, Elizabeth P. Blankenhorn, John P. Mordes

**Affiliations:** ^1^Program in Bioinformatics and Integrative Biology, University of Massachusetts Medical School, Worcester, MA 01605, USA; ^2^Department of Microbiology and Immunology, Center for Immunogenetics and Inflammatory Diseases, Drexel University College of Medicine, Philadelphia, PA 19129, USA; ^3^Children's Hospital Oakland Research Institute, Oakland, CA 94609, USA; ^4^Department of Medicine, University of Massachusetts Medical School, Worcester, MA 01605, USA

## Abstract

Type 1 diabetes (T1D) is a T cell-mediated disease. It is strongly associated with susceptibility haplotypes within the major histocompatibility complex, but this association accounts for an estimated 50% of susceptibility. Other studies have identified as many as 50 additional susceptibility loci, but the effect of most is very modest (odds ratio (OR) <1.5). What accounts for the “missing heritability” is unknown and is often attributed to environmental factors. Here we review new data on the cognate ligand of MHC molecules, the T cell receptor (TCR). In rats, we found that one allele of a TCR variable gene, V**β**13A, is strongly associated with T1D (OR >5) and that deletion of V**β**13+ T cells prevents diabetes. A role for the TCR is also suspected in NOD mice, but TCR regions have not been associated with human T1D. To investigate this disparity, we tested the hypothesis *in silico* that previous studies of human T1D genetics were underpowered to detect MHC-contingent TCR susceptibility. We show that stratifying by MHC markedly increases statistical power to detect potential TCR susceptibility alleles. We suggest that the TCR regions are viable candidates for T1D susceptibility genes, could account for “missing heritability,” and could be targets for prevention.

## 1. Introduction

Type 1 diabetes (T1D) is a T cell-mediated autoimmune disease that afflicts a million persons in the USA [1, 2]. It is a polygenic disorder resulting from the interaction of multiple gene variants [3] and environmental factors [4]. No approved methods are currently available for its prevention or reversal [5]. Most interventions targeted at curing human T1D have focused on either “secondary” or “tertiary” prevention, that is, treating individuals who either have the disease or are at risk based on family history and autoantibody titers [5]. To date, no intervention has achieved the degree of success required for clinical adaptation [6]. 

New strategies for primary prevention in susceptible individuals would be advantageous, attacking the problem before it starts or at its earliest stages [7]. Primary prevention, however, requires accurate predictive genetic tools. Treatment of individuals who would have remained diabetes-free poses serious pragmatic and ethical issues. 

The major genetic loci for diabetes susceptibility are within the human leukocyte antigen (HLA) region, specifically those encoding HLA-DR and DQ antigens, with a less significant independent contribution from HLA class I genes [8–10]. Several high-risk HLA class II haplotypes account for ~40% of the predisposition to T1D, with an odds ratio of ~6.8, but accounting for the remaining 60% is an unresolved problem [3]. The insulin genes VNTR, *PTPN22*, and *CD25* are associated with odds ratios >1.5, and rare alleles of *IFIH1* have an odds ratio (OR) near 0.5 [11]. More than 40 non-MHC genes/regions, most involved in immune responses, have statistically significant associations, but with OR <1.5 [11, 12]. 

Unfortunately, although low-resolution HLA-genotyping will identify most individuals at risk for T1D, only 1/15 or ~7% of individuals with one of the highest risk HLA genotypes (known as “DR3/DR4”) will actually become diabetic [13]. Additional genetic knowledge has not yet significantly improved prediction. An early estimate of sibling relative risk (*λ*s) for T1D was estimated to be quite high at 15 [14]. Predictions of T1D, incorporating both HLA and all currently known loci, generate a *λ*s of only 5 [3, 15], although a recently reported strategy based on combining multiple risk alleles appears to hold promise [16].

## 2. The TCR and “Missing Heritability”

A possible explanation for our inability to predict T1D accurately based on genotyping is the effect of environmental perturbants [21]. There is clear seasonal and geographic variation in the onset of T1D [22], and miniepidemics of the disease have been documented [23]. Viral infection is thought to be the most likely perturbant, and it remains a topic of intense investigation [24]. Although there is no good evidence for direct infection of pancreatic beta cells, the immune response to infection might easily provoke disease onset in genetically predisposed individuals. Most of the genes and loci identified by genome-wide association study (GWAS) analyses of T1D are involved in immune responses [11], and the interaction of random infection with such genes (“environmental genetics”) is a plausible way to account for the “missing heritability.”

**Figure 1:**
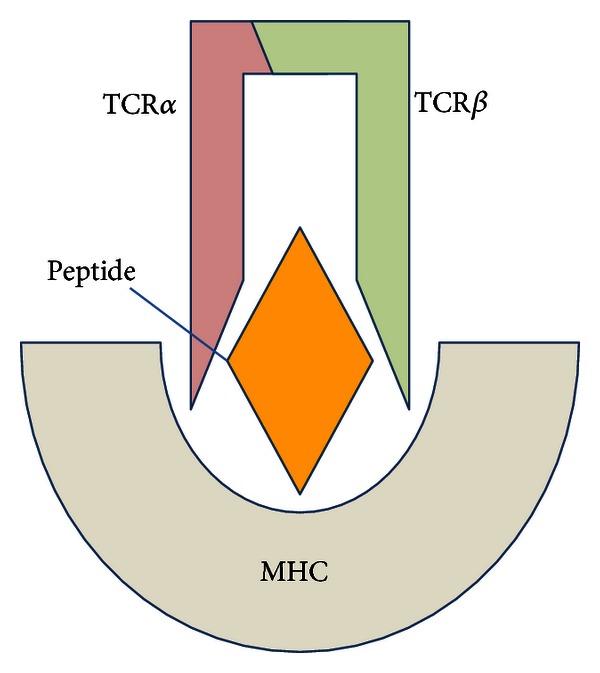
The trimolecular TCR-pMHC complex that is fundamental in T1D susceptibility.

We would like to suggest, however, that there may be an overlooked genetic element that has not been detected for technical reasons, specifically the genome-encoded parts of the T cell receptor (TCR). The TCR is the cognate partner of major histocompatibility complex (MHC) molecules in the peptide-MHC (pMHC) unit (Figure 1), and T1D is clearly a T cell-mediated disease. Nonetheless, there is very little evidence that germline TCR haplotype is important in susceptibility to T1D. There are, however, linkages to TCR in human autoimmune diseases other than T1D. TCR genotype has been implicated in multiple sclerosis (MS) [25]. There are also well-documented associations of TCR genotype with other forms of autoimmunity including Sjogren's syndrome [26, 27] and narcolepsy [28–30], which has a *TCRA* bias.

Of course, because approximately 10^15^ V-(D)-J recombined TCRs are possible, it is not surprising that a role for germline-encoded TCR usage, with far less diversity than the recombined genes, has met with skepticism. New data, however, suggest that the genome-encoded TCR is likely to play a critical, previously unrecognized role in the pathogenesis of T1D. Here we review our data that point to a role for genome-encoded TCR susceptibility to T1D and then present new quantitative analyses that attempt to account for the failure of previous studies to detect such an effect.

## 3. Evidence from the Rat

### 3.1. Gene Mapping

Type 1-like autoimmune diabetes, both spontaneous and inducible, is relatively common among inbred rat strains that, like humans, express a high-risk class II MHC haplotype. In rats, this is designated* RT1B/Du* [19, 31, 32]. We have previously reported that *Iddm14 *(formerly designated *Iddm4*) is a dominant non-MHC susceptibility locus important for both spontaneous and induced autoimmune diabetes in multiple rat strains [18, 33–38]. 

Studies of *Iddm14* in eight *RT1B/Du* rat strains led to the identification of a susceptibility haplotype in the* Tcrb-V* locus [18]. Sequencing and single nucleotide polymorphism (SNP) haplotype mapping revealed that 6 rat strains susceptible to diabetes (KDP, BBDR, BBDP, LEW.1WR1, LEW.1AR1-iddm, and PVG-RT1u) all share one allele of the beta chain variable region gene *Tcrb-V13 *(designated *Tcrb-V13S1A1*) [20]. Three rat strains that are resistant to, or confer resistance to, diabetes in genetic studies, all express different alleles, either *Tcrb-V13S1A2* in the case of BN and WF rats, or *Tcrb-V13S1A3P* in the F344 rat [20]. These polymorphisms are of interest because the *Tcrb-V13S1A1* gene product, designated V*β*13a, is used more by CD4+ than CD8+ cells [20]. Taken in the context of additional data available from studies of rat T1D, our findings suggest that it is the combination of MHC and TCR that in large measure determines susceptibility to T1D in the rat. As summarized in Table 1, only those rats that express both RT1B/Du and V*β*13a are highly susceptible to T1D. In the absence of V*β*13a, rats with a high-risk MHC are relatively resistant to T1D, and in the absence of RT1B/Du essentially no rats develop autoimmune diabetes.

These genetic observations, consistent with a critical role for germline TCR usage in T1D in the rat, led us to hypothesize that allele-specific TCR targeting could substantially prevent disease. This hypothesis was confirmed in multiple model systems, described below.

### 3.2. Depletion of V*β*13+ T Cells Prevents Poly I:C-Triggered T1D

LEW.1WR1 rats have a normal immunophenotype and develop T1D spontaneously at a rate of 2.5% and after treatment with polyinosinic: polycytidylic acid (poly I:C, a TLR3 and IFIH1 ligand) at a rate of 90–100% [39]. After documenting that anti-V*β*13 monoclonal antibody (mAb) reduces the number of V*β*13+ T cells *in vivo* by about 60%, we compared diabetes frequency in rats treated with either anti-V*β*13 mAb or control mouse anti-human OKT8. A second trial compared diabetes frequency in rats treated with anti-V*β*13 mAb, depleting anti-V*β*16 mAb, or vehicle. Diabetes frequency in rats treated with poly I:C and anti-V*β*13 mAb was 10% (2/20). In contrast, diabetes frequency in controls averaged 85% (34/40, *P* < 0.001) [17]. Histologic study showed significantly less insulitis and nearly complete preservation of beta cell insulin in animals treated with depleting anti-V*β*13 [17].

### 3.3. Depletion of V*β*13+ T Cells Prevents Virus-Triggered T1D

We also tested a model of triggered diabetes induced by viral infection. Rats were given a small priming dose of poly I:C followed by infection with Kilham rat virus (KRV). The priming dose of poly I:C is by itself nondiabetogenic but increases the penetrance of virus-triggered diabetes from ~40% to ~100% [40]. Diabetes frequency in anti-V*β*13-treated rats was 30% (3/10) as compared with 80% (8/10, *P* = 0.03) in both anti-V*β*16 mAb treated animals and untreated controls.

### 3.4. Depletion of V*β*13+ T Cells Prevents Spontaneous T1D

BBDP rats develop spontaneous T1D at a rate of 60–90% [31]. We treated cohorts of BBDP rats with vehicle, anti-V*β*13 mAb, or anti-V*β*16 mAb. Treatment with anti-V*β*13 mAb through 100 days of age completely prevented diabetes, whereas diabetes occurred in vehicle injected and anti-V*β*16 mAb treated rats at rates of 40% and 70%, respectively (*P* < 0.01). Among rats still nondiabetic at the end of the experiment, there was substantial “simmering” insulitis in rats treated with anti-V*β*16 mAb or vehicle [40].

These prevention studies were supplemented by additional immunological data showing a critical role for V*β*13+ T cells early in T1D pathogenesis. 

### 3.5. CD4+V*β*13+ T Cells Are Abundant in Islets Early in the Disease Process

The animal models of “triggered” T1D that we use have well-defined kinetics and relatively rapid onset. This allows us to harvest islets from animals very early during disease onset and study the infiltrating inflammatory cells. By day 5 CD4+V*β*13+ T cells are remarkably abundant in the prediabetic islet [40], reaching a peak on day 10, when overt diabetes is first detectable.

### 3.6. V*β*13/J*β* mRNA Transcripts in Prediabetic Islets Are Skewed

Upon cloning the V*β*13+ transcripts from early prediabetic islets, we observed significant skewing of the TCR*β* repertoire, with pauciclonal expansion of V*β*13-CDR3 sequences from islet T-cells compared to a high diversity of V*β*13-CDR3s in spleen. These data suggested that antigen-specific expansion of V*β*13+ T cells occurs in the islets of prediabetic rats. We also observed skewed TCR-J*β* usage in islet-infiltrating V*β*13+ T cells, with overrepresentation of J*β*1.3 and underrepresentation of J*β*2.1 relative to peripheral T cells. Spleen V*β*13+ T cells from poly I:C treated and untreated rats display skewing of individual J*β* segments. In addition, the representation of different J*β* segments in V*β*16+ T cell transcripts was not skewed in the islets or in the periphery. These results strongly support a role for V*β*13+ T cells in the early recognition of antigen in islets. In addition, we showed that the TCR-V*α*5 repertoire is skewed among islet homing, sorted V*β*13+ T cells. This is exciting because TCR-V*α*5D-4 is frequently used in the mouse T-cell response to islet antigen and recognizes insulin B:9-23 [41, 42]. Collectively, these data indicate that an oligoclonal V*β*13 response to pancreatic beta cells exists early in progression to autoimmune diabetes. 

## 4. Evidence from the NOD Mouse

In the NOD mouse, Abiru et al. have observed a dramatic TCR *α* chain restriction (predominantly V*α*5) in the recognition of insulin autoantigen [44]. Retrogenic NOD strains expressing V*α*5D-4 *α* chains with many different CDR3 sequences show that even those derived from TCRs recognizing islet-irrelevant molecules develop anti-insulin autoimmunity [41]. The germline encoded V*α*5D-4 T cell receptor targets a primary insulin peptide in NOD mice [41]. In addition, induction of insulin autoantibody production by helper T cells bearing V*α*5D-4 *α* chains can be abrogated by the mutation of two amino acid residues in CDR1 and CDR2 sequences of *TRAV5D-4*. *TRAV13-1*, the human ortholog of murine *TRAV5D-4*, was also capable of inducing *in vivo* anti-insulin autoimmunity in the NOD mouse [41].

Nevertheless, the *V*α** locus has never been detected in mouse linkage studies to discover T1D genes. TCR-V*α*5D-4 is polymorphic in mice, with 5 identified alleles differing by only 2–4 amino acids, but it also belongs to a family of paralogous genes with >92% homology (International ImMunoGeneTics information system or IMGT, http://www.imgt.org/). There is a paralog in B6 that has the same CDR1 and CDR2 as the important NOD paralog, *VA*5*D*4*04. There are two non-CDR amino acid differences in this B6 paralog, which by their position are unlikely to contribute to the trimolecular complex [45]. This means that both NOD and B6 strains could transmit an effective diabetogenic allele of a TCR-V*α*5D-4, and thus there would be no apparent linkage to the *V*α** locus (resulting in the *Idd* designation) in a backcross or F2 [46]. 

A separate issue is whether a *V*α** (and TCR-V*α*5D-4 in particular) selectively recognizes a permissive diabetogenic MHC molecule. A hint that this might occur is found in the mouse, where paralogs/alleles of TCR-V*α*5D-4 likely influence differential selection on polymorphic MHC haplotypes. This is supported by the finding that the expressed repertoire of TCR-V*α*5 paralogs in mice has a different frequency distribution in the periphery of mice with different MHC-II haplotypes [47]. In contrast, *Iddm14* (TCR-V*β*13-A1) was discovered in rats in a linkage study because this TCR allelic polymorphism exists among multiple strains bearing the same high-risk MHC-II haplotypes.

## 5. Data That Point toward a Mechanism Explaining the Role of TCR Genotype

### 5.1. Sequence Data in the Rat

**Figure 2:**
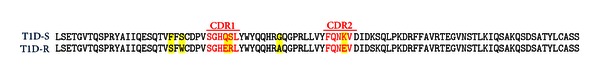
Nonsynonymous amino acid sequence alignments of beta chain CDR1 and CDR2 regions of V*β*13a, found in T1D susceptible (T1D-S) rats, and V*β*13b, which is found in T1D resistant (T1D-R) rats [17, 20]. Both the CDR1 and CDR2 regions (red) exhibit differences (all indicated in yellow). V*β*13a is encoded by *Tcrb-V13S1A1* and has been found in the BBDR, BBDP, LEW.1WR1, LEW.1AR1-*iddm*, KDP, and PVGRT1u strains, all of which are T1D susceptible; V*β*13b is encoded by *Tcrb-V13S1A2* and is found in T1D resistant WF and BN rats [17]. Another allele, *Tcrb-V13S1A3P*, is a pseudogene found in the resistant F344 rat [17, 20].

The gene products of *Tcrb-V13S1A1 *and *Tcrb-V13S1A2* encode different amino acid sequences for both the CDR1 and CDR2 regions of the beta chain [20]. This polymorphism distinguishes WF and other T1D-resistant strains from BBDR, BBDP, KDP, and LEW.1WR1 T1D-susceptible strains, all of which share the same class II MHC [18]. CDR1 and CDR2 sequences are encoded within each *Tcrb-V* allele and are not altered by the combinatorial processes that create the CDR3 regions of the TCR (Figure 2).

The V*β*13 sequences shown in Figure 2, which differentiate our susceptible and resistant alleles of *Tcrb-V13, *differ in both CDR1 and CDR2 and are consistent with emerging data on structural elements of the TCR-pMHC synapse that affect not only peptide recognition, but also binding affinity and peptide registration [48]. 

### 5.2. Structural Analyses

Crystal structures of the TCR-pMHC reveal the importance of the CDR1 and CDR2 regions in the human immunological synapse (recently reviewed in [48]). It is well accepted that CDR1 and CDR2 are critical for T cell-MHC restriction [49], and new data reveal how they interact with MHC helices to produce unanticipated and potentially important effects. In one study of the TCR “energetic landscape” it was noted that CDR1 and CDR2 loops act in a major way to stabilize the ligated CDR3 loops [50]. 

Another recent crystallographic study was designed specifically to address the question of whether shared germline contacts within the TCR-pMHC would persist despite distinct CDR3-peptide contacts in the model system, and they do [51]. The authors concluded that, “…a TCR utilizing entirely distinct chemistries to recognize different peptides exhibits highly persistent germline-mediated contacts.” 

Studies by Sethi et al. have reported the crystal structure of a TCR from a patient with multiple sclerosis that engages its pMHC ligand in an unusual manner [52]. The TCR is bound in a highly tilted orientation that prevents interaction of the TCR-*α* chain with the MHC class II *β* chain helix. In this structure, only a single germline-encoded (i.e., CDR1 or CDR2) TCRV*β* loop engages the MHC protein. Furthermore, the reduced interaction surface with the peptide may facilitate TCR cross-reactivity. 

Finally, a very recent study shows biased TCR usage against HLA DQ8-restricted gliadin peptides in persons with celiac disease [53]. These new data show that TCR usage biased to *TRBV*9*01 underpins the recognition of HLA-DQ8-*α*-1-gliadin. More importantly for our hypothesis, they show that “*all* CDR*β* loops (not just CDR*β*3) interact with the gliadin peptide.” They proved that “…Leu37*β* from the CDR1*β* loop, and Tyr57*β* from the CDR2*β* loop are the “hot spot” residues underpinning the SP3.4 TCR-DQ8-glia-*α*1 interaction providing a basis for the *TRBV*9*01 bias.” This is precisely what our rat data predict to be true in T1D (which is often comorbid with celiac disease). Interestingly, rat V*β*13 is polymorphic in the analogous CDR1*β* position 37 “hot spot” described for celiac disease, strengthening the notion that allelic polymorphism in V*β*13 may influence pMHC interaction.

## 6. TCR Allelism in the Human Genome

A number of factors could affect investigations of the role of genome-encoded TCR sequences in human T1D. One is the issue of paralogs, which we discuss using the NOD V*α*5D-4 as an example. Like the NOD, humans could also have multiple paralogs that are capable of binding insulin, and thus, some linkage studies would not find a T1D gene in the *V*α** region if the parents each had one or more suitable *V*α** paralogs. However, GWAS evaluates many more individuals than a linkage study does, and would have more power to detect those individuals who do not possess a suitable allele of a *V*α** chain paralog that detects insulin autoantigen. Deep sequencing has not been performed on the *V*α** and *V*β** regions in large numbers of people, so it is premature to suggest that such individuals do not exist. To date, however, the human *TCR*α** locus does seem significantly less complex than in rodents due to rat- and mouse-specific gene duplication events and/or human specific gene convergence (IMGT), so that human paralogs might in fact not complicate the genetic identification of a T1D gene in the *V*α** region. 

**Figure 3:**
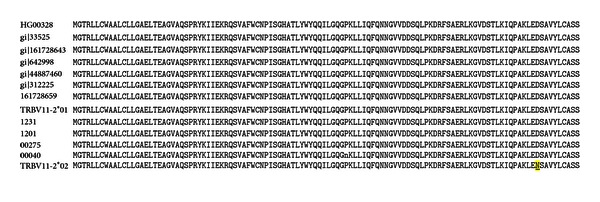
Genomic TRBV11-2 DNA sequences from Caucasian samples in the 1000 genomes database (including HG00328 (and HG00361, HG00320, HG00111, HG00310, HG00247, HG00256, HG00231, HG00127, HG00103, HG00117, HG0032, not shown)), five expressed sequences from the dbEST (indicated with gi numbers), two alleles of TRBV11-2 from IMGT, and four of the sequences we obtained at Drexel (1231, 1201, 00275, 00040) were translated and compared. Only TRBV11-2*02 shows any nonsynonymous change (D105N, underlined, yellow highlight), and it is substantiated by one transcript (M33235). The D105N change (which is residue 98 using IMGT numbering) forms a salt bridge with a positive residue (Arg75) in the V*β* domain but is away from the pMHC (see Supplementary Figure 1 available online at http://dx.doi.org//10.1155/2013/737485). Accordingly there could be some functional/structural consequence of that SNP, but given its location (not in any CDR region) and conservative nature (Asp to Asn) it is unlikely that this is a functionally significant change. There is, however, no proof that this is the case. Nucleotide substitutions reflecting known SNPs (rs183490568, rs149749379, rs148941368, rs139187012, rs76976752, rs34112565, rs17163285, rs7777952, rs17281, rs17163283, rs17280, rs11505614, rs57147993, rs10375465, and rs17279) were commonly observed among these sequences. Using these SNPs, four haplotypes (two homozygous and two heterozygous) were observed among the four Drexel sequences.

Another factor that could affect investigations of the role of genome-encoded TCR sequences in human T1D is the possibility of extensive undocumented allelism. To investigate this possibility, we performed a preliminary study and analyzed one TCR isotype, *TRBV11-2*, in detail as proof of principle; it was selected because it is the human homolog of rat *Tcrb-V13*. From the 1000 Genomes database [57], we retrieved several *TRBV11-2* sequences in which clear null alleles were present (some with open reading frames but no possibility of use in a TCR, as they did not have proper predicted cysteines or tryptophans at key residues ([57], (http://www.imgt.org/)). Among the remaining 1000 Genomes Caucasian sequences were multiple examples bearing the most common SNP variants, which supports the expected average minor allele frequency (MAF) of several of the SNPs in this region (0.21–0.43 among Caucasians, 1000 Genomes database). We sequenced a small number of samples at Drexel, to determine the level of polymorphism in this TCR element. The predominant *TRBV11-2* allele among our sequences is supported by numerous ESTs and cDNAs (Figure 3). Interestingly, the UCSC database (hg19) [58] has a reference sequence for *TRBV11-2* with a stop codon in the leader sequence, but all four of these *TRBV11-2* genes have an open reading frame throughout the entire gene. The low read depth of sequencing, and the likely heterozygosity in the 1000 Genomes database, can contribute to ambiguous calls, leading us to question all but the most substantiated alleles of 11-2. Therefore, we searched the NCBI EST/cDNA databases for known transcripts with homology to *TRBV11-2* (using BLAST), and these confirm the major alleles we found by sequencing. Our results compare with only two amino acid substitution alleles recognized previously in IMGT. 

To generate a comprehensive analysis of genomic TCR effects in T1D, it will be necessary to collect alleles from each chromosome of heterozygous individuals. This information is missing from any available database, as most report a single sequence from each “person” in the cohort studied. As it is likely that people are polymorphic for TCR V*β* regions, we expect that many will be heterozygous. Thus, data are needed to fill the gap in our knowledge about variation in *TRAV* and *TRBV* as well as to contribute information on linkage disequilibrium (LD) between the many different isotypes within the TCR genomic complexes.

## 7. Molecular and Analytical Basis for Identification of Human TCRs Involved in T1D

### 7.1. Identifying and Mapping Known Human TCR Polymorphisms

To examine the possibility of germ-line human TCR variants having a functional impact on the immune repertoire and a potential impact on autoimmunity and T1D in particular, we extended the analysis of polymorphism in *TRBV* and compiled a comprehensive set of nonsynonymous polymorphisms in the human *TRAV* and *TRBV* genes in regions known to contact the pMHC (Table 2) [56]. In addition to the CDR1 and CDR2 loops, we included the TCR N-termini and HV4 loops, which often have 1-2 residues contacting pMHC [59, 60]. Our results (Table 2) indicate that there is a notable degree of polymorphism in both the TCR*α* and TCR*β* chains, in regions with direct impact on pMHC binding. This was also noted in a previous study of human *TRAV* gene diversity where the authors found that nucleotide diversity was “substantially higher in the CDRs versus the FRs (framework regions)” [56]. This diversity is observed in a total of 19 positions and 22 genes, including the *TRBV9* gene, which, as noted above, is implicated in celiac disease [53]. 

It is not certain whether the particular polymorphism in TRBV9 (Q55H) plays a key role in recognition of the gliadin-HLA-DQ8 celiac disease antigen (see above), though a structural and biophysical study of the TK3 TCR, which is encoded by the *TRBV9* gene, found that this particular variant affected the electrostatic makeup and structure of pMHC recognition for HLA-B*35:01 and an EBV peptide [61].

**Figure 4:**
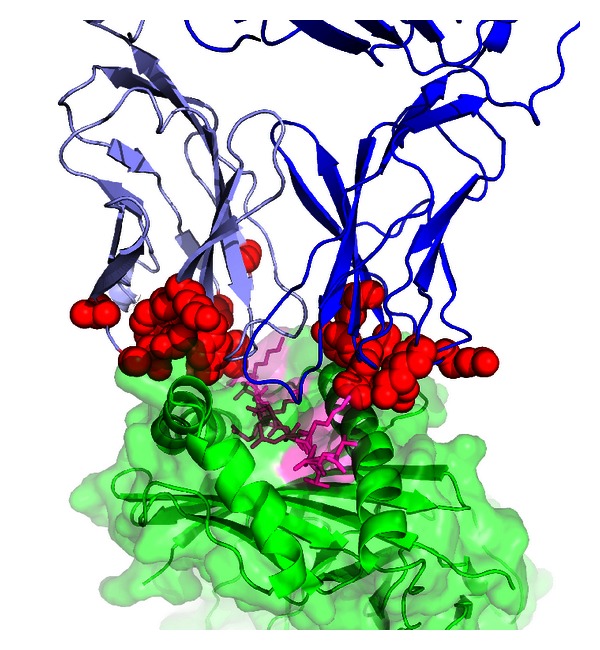
TRAV and TRBV polymorphic positions shown on a TCR/pMHC complex structure. Polymorphic positions are red, TCR*α* is light blue, TCR*β* is blue, peptide is magenta, and MHC is green. Structure shown is HA1.7 TCR/HA peptide/HLA-DR4 (Protein Data Bank [43] ID 1J8H). The figure was generated using PyMOL (http://www.pymol.org/).

To provide a structural context for these TCR sequence polymorphisms, we mapped their positions onto the structure of a complex of a TCR with a Class II MHC and peptide (Figure 4). While not all TCRs exhibit the same docking geometry, this particular complex [62] has a typical pMHC docking angle (49°) and is, thus, approximately representative of a variety of known complexes [63]. These polymorphisms in germ-line encoded TCR genes have a clear potential to impact the pMHC recognition, and, as the figure indicates, primarily via interaction with MHC helices. Additionally, a subset of these variants (including TCR*β* position 55 in the case of the TK3 TCR [61]) have the potential to directly interact with the peptide as well. It is worth noting that additional positions outside of those analyzed here can potentially impact pMHC binding, given that TCRs are known to exhibit long-range energetic effects [64, 65] and long-range dynamic coupling between distal TCR sites [66]. It should also be noted that Table 2 is very likely an incomplete representation of TCR polymorphisms, and next generation sequencing of T cells from diseased populations, in conjunction with larger-scale studies of exome sequencing (including the 1000 Genomes project [67]), should yield valuable data on TCR polymorphisms and insights into their impact on autoimmune diseases like T1D and control of infection. 

### 7.2. Improving Detection via Targeted Genotyping and Next Generation Sequencing

The question remains, then: why have such putative TCR alleles not been detected in GWAS studies? To address this issue, we performed a quantitative analysis. Assume that some *TCRV* allele, call it “*Vx*,” is important for T1D in individuals with a particular HLA-DRB1 allele (e.g., HLA-DRB1*03:01, which will be referred to here as DR3). We base this assumption on our data showing that V*β*13a is important for T1D in MHC Class II *RT1Bu* rats. Our remaining analysis is as follows.

#### 7.2.1. Stratification by DR Allele

The ability to detect a causative “*Vx*” TCR allele may be dramatically improved by stratifying disease population by HLA-DR allele. Here we consider stratification by HLA diplotype (rather than presence of one haplotype alone), based on the impact of trans-encoded HLA proteins on T1D susceptibility and immune function [8, 68], as observed for the high-risk trans-encoded HLA-DQA1*05:01/DQB1*03:02. Additionally, this will control for the potential confounding effects of another HLA allele whose interaction with a different TCR could confer either additional susceptibility or resistance. However, such analysis (and stratification) can be applied for single alleles (i.e., the percentage of the T1D population with at least one copy of an allele), though haplotype frequencies (as given in Table 2 of Erlich et al. [8]) would not suffice. This is because they provide overall frequency of haplotypes within all diploid genomes (not distinguishing between homozygous and heterozygous).

As an example, we will focus on one of the high-risk HLA alleles, DR3, but the argument can be applied to any allele of interest. The frequency of DR3/3 homozygotes in T1D patients is 7.6% [8]. Assuming that the “*Vx*” allele is relevant in the context of HLA-DR3/3 and not the remainder of the HLA-DR allele combinations, the odds ratio seen for all T1D subjects (OR_All_) is scaled accordingly from the odds ratio of “*Vx*” within the DR3/3 T1D population (OR_DR3/3_) by the DR3/3 frequency:

(1) $$\begin{array}{c} {\text{OR}}_{\text{All}}=0.076*{\text{OR}}_{\text{DR}3/3}+0.924. \end{array}$$

**Figure 5:**
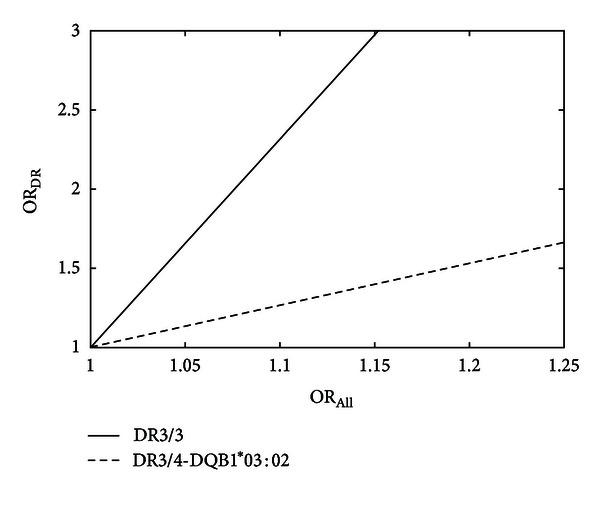
Calculated improvement in odds ratio (OR) when stratifying by HLA-DR-DQ genotype (OR_DR_) versus analyzing all T1D patients (OR_All_) for detection of a putative risk TCR allele. Shown are data for homozygous HLA-DR3/3 and the high-risk DR3/4-DQB1*03:02 diplotype.

A comparison of stratified versus unstratified ORs is shown in Figure 5, for both DR3/3 and another diplotype (*HLA*-*DR*3/4-*DQB*1*03:02), which is more frequent in T1D (38% of the T1D population with European ancestry [8]) and contains the high-risk trans-encoded *HLA*-DQA*05:01/*HLA*-DQB*03:02 noted above. Even for a relatively modest value of OR_All_ (1.1, which is below the level of most identified genes in GWAS studies [11]), the OR for DR3/3 becomes appreciably higher (2.3) and somewhat higher (1.27) for *DR*3/4-*DQB*1*03:02. Given these odds ratios, for a study with 2000 patients, 2000 controls and background allele frequency of 10%, power to detect the risk TCR allele increases from 16% to 75% for *DR*3/4-*DQB*1*03:02, and to 100% for *DR3/3* (G*Power version 3.1, Fisher's two-tailed exact test, *α* = 0.05). This implies that for previous studies that did not include stratification by HLA alleles, detection of a “*Vx*” allele may have been confounded by dilution of the odds ratio.

This analysis helps explain why a TCR *α* chain locus associated with autoimmune narcolepsy was identified using GWAS [29]. That study was stratified by default because a single HLA haplotype (*DR* *B*1*15:01-*DQB*1*06:02) is seen in 90% of cases [69]. That study did not identify the specific allele of a TCR that confers narcolepsy, though one might be identified using next generation sequencing (NGS).

#### 7.2.2. NGS versus GWAS Array Coverage

Traditionally, analysis of polymorphisms in T1D has involved genome-wide association studies (GWASs), which rely on arrays with defined sets of probes, to infer the genotype in different regions of the locus. An analysis of linkage disequilibrium (LD) at the human *TRAV* locus found that it is highly variable, leading the authors to conclude that, “even with relatively dense coverage, it is unlikely that a genotyping strategy (as opposed to a resequencing strategy) will provide adequately dense coverage extending into the *V* genes” [56]. We examined this contention further using a dataset of *TRAV* and *TRBV* SNPs downloaded from the UCSC genome browser [58] (dbSNP [70] build 130), selecting only SNPs found within TRAV and *TRBV* exons from IMGT reference sequences [55]. This resulted in a total of 322 SNPs (158 in *TRAV* exons and 164 in TRBV exons). We used the SNAP web server [71] to calculate the degree of correlation (*r* ^2^) of these SNPs with three arrays used in previous studies of GWAS in T1D (Affymetrix GeneChip 500K, Illumina 550K Infinium and Affymetrix SNP Array 6.0) [72] and found average *r* ^2^ of approximately 0.3, and approximately 25% of SNPs with *r* ^2^ of 0.8 or higher. This coverage is somewhat lower than observed for common genome-wide SNPs from European ancestry for the Affymetrix GeneChip 500 (commensurate with values for the less common SNPs with MAFs of 1–5%) [73], but since these SNPs were not classified by frequency it is not possible to conclude that *TRAV/TRBV* coverage is lower than the rest of the genome, or determine the coverage of SNPs above a certain frequency threshold. Regardless, in light of the previous findings of variable LD at these loci noted above, these data support the notion that average *r* ^2^ for *TRAV* and *TRBV* SNPs is markedly lower than 1. As correlation is directly proportional to power of GWAS studies [74], it follows that there is likely at least some reduction in power to detect putative TCR alleles, reinforcing the strategy of NGS to genotype TCR polymorphisms. 

## 8. Conclusion

The striking observations that have recently been made in the NOD mouse and in multiple strains of rats that are used to model T1D strongly suggest that elements of the TCR, encoded at the level of the genome and not subject to V-(D)-J recombination, may play a critical role in T1D susceptibility in an MHC-dependent fashion. Assuming these animal model systems are reliable, the likelihood is high that the germline TCR regions are also important for susceptibility to human T1D. This is not a new idea, but no supporting data have been reported to date. However, our quantitative analyses show that cogent statistical flaws in previous approaches could account for the negative findings. If an HLA-dependent TCR susceptibility paradigm can be identified for human T1D in the future, it could open the path to new ways of preventing the disease, either by targeting specific alleles of the TCR with deletion approaches or by using small molecules to interfere with the specific TCR-pMHC synapses that lead to the disease. 

## Supplementary Material

The Supplemental Figure shows the position of the D105N polymorphism (discussed in Figure 3 of the main text) in the crystal structure of the CF34 TCR (which is encoded by the TRBV 11-2∗01 allele) in complex with HLA-B∗0801 and a viral peptide (Protein Data Bank ID 3FFC). The position in question (which is residue 98 using IMGT numbering and in the PDB entry) is shown in red and forms a salt bridge with a positive residue (Arg75; shown as cyan sticks) in the V*β* domain but it is away from the pMHC (approximately 22 Å) and CDRs. TCR *α* chain is light blue, *β* chain is blue, peptide is magenta, and MHC is green. The supplemental figure was generated using PyMOL (http://www.pymol.org/).

## Figures and Tables

**Table 1:** Type 1 diabetes frequency in rats as a function of MHC and TCR genotypes.

TCR	MHC	Diabetes	Strains
V*β*13a present	*RT1B/Du *	High susceptibility to diabetes	BBDP and BBDR
			LEW.1WR1 and LEW.1AR1-iddm
			KDP
			PVG.*RT1u *
V*β*13a absent	*RT1B/Du *	Low penetrance of T1D	WF
V*β*13a present	Non-*RT1B/Du *	No T1D	Many

Coassociation of Class II MHC haplotype and TCR usage in T1D in rats [17–19].

**Table 2:** Previously identified polymorphisms in *TRAV/TRBV* genes near the pMHC interface.

TCR Location^1^	Polymorphism(s)^2^	Genes^2^
N-term	N2D	*TRAV9-2 *
CDR1*α*	V27M, G29V, ***G29R***, ***N30S***, P30E, P30Q, N31D, ***Y32S ***	*TRAV36, TRAV12-2, TRAV8-4, TRAV14-1, TRAV38-1, * ***TRAV20 ***
CDR2*α*	F55S, ***Q56E***, A57G, ***V57M***, S58T, ***T58I***, A59G, ***K59E***, Q61E	*TRAV12-2, TRAV1-1, TRAV8-4, TRAV14-1, * ***TRAV25, TRAV8-7, TRAV26-2, TRAV38-1 ***
CDR1*β*	A30V, N30E	*TRBV7-7, TRBV6-6 *
CDR2*β*	Q55H, Q57H, V57I, D58N, G60D, S60C, Q60H, L61I	*TRBV9, TRBV19, TRBV30, TRBV15, TRBV20-1, TRBV10-1, TRBV3-1 *
HV4*β*	G84E	*TRBV7-2 *

^1^Region of the TCR variable domain tertiary structure. CDRs are as defined by Lefranc et al. [54], with CDR2 extended by one residue at the N-terminus to account for pMHC contacts with this position. ^2^From IMGT [55], as well as additional data from Mackelprang et al. [56] (in bold italics). IMGT TCR residue numbering used.
