# Supplementary material for: The Missing Heritability in T1D and Potential New Targets for Prevention
Source: J Diabetes Res. 2013 Mar 13;2013:737485. doi: 10.1155/2013/737485 (PMC3647582; doi:10.1155/2013/737485)
Supplement: Supplementary file 1 — The Supplemental Figure shows the position of the D105N polymorphism (discussed in Figure 3 of the main text) in the crystal structure of the CF34 TCR (which is encoded by the TRBV 11-2∗01 allele) in complex with HLA-B∗0801 and a viral peptide (Protein Data Bank ID 3FFC). The position in question (which is residue 98 using IMGT numbering and in the PDB entry) is shown in red and forms a salt bridge with a positive residue (Arg75; shown as cyan sticks) in the Vβ domain but it is away from the pMHC (approximately 22 Å) and CDRs. TCR α chain is light blue, β chain is blue, peptide is magenta, and MHC is green. The supplemental figure was generated using PyMOL (http://www.pymol.org/). [file 737485.f1.pdf]

Supplemental Figure 1

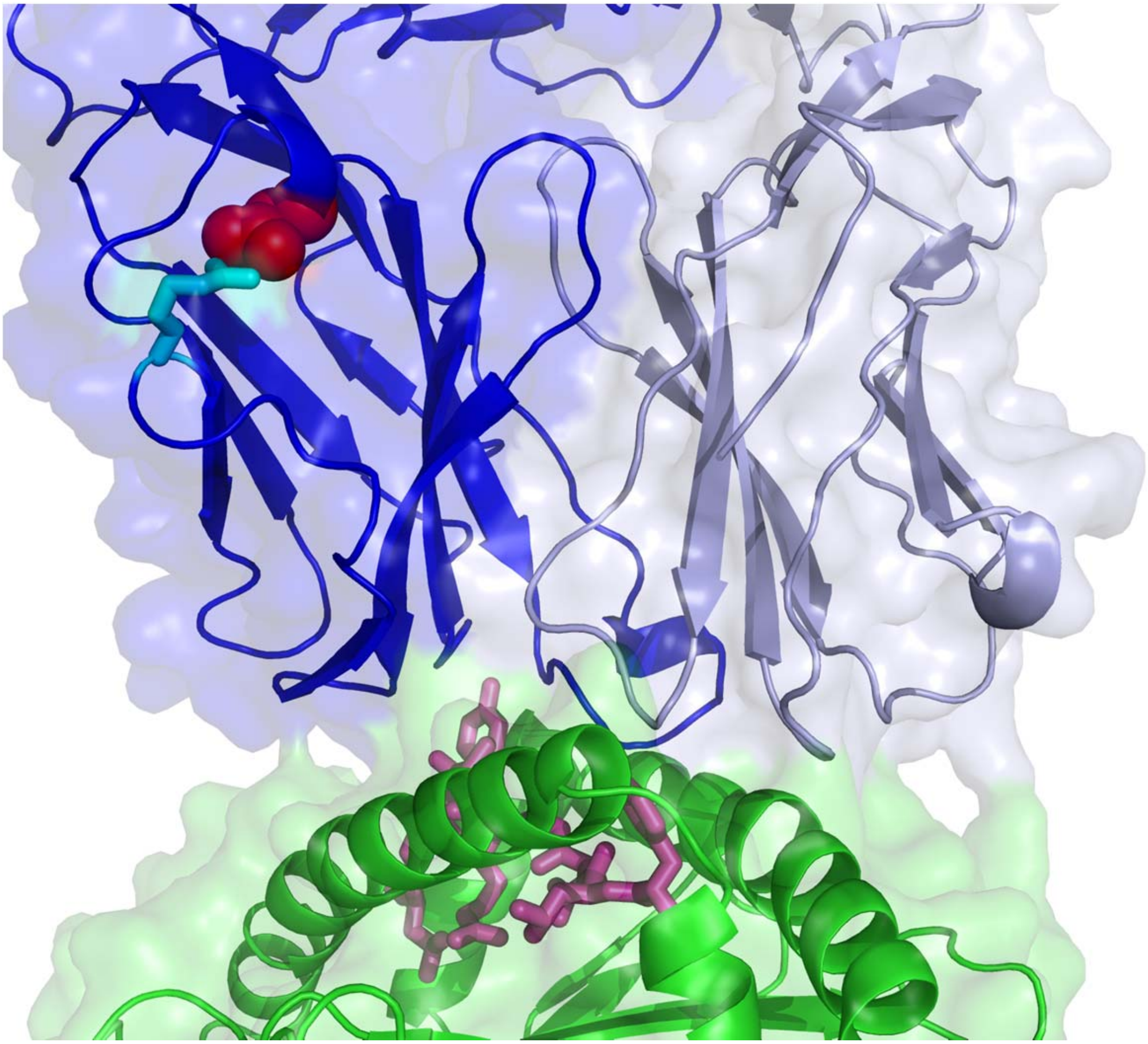

Legend to Supplemental Figure 1: Position of the D105N polymorphism (discussed in Figure 3 of the main text) in the crystal structure of the CF34 TCR (which is encoded by the TRBV 11-2*01 allele) in complex with HLA-B*0801 and a viral peptide (Protein Data Bank ID 3FFC). The position in question (which is residue 98 using IMGT numbering and in the PDB entry) is shown in red and forms a salt bridge with a positive residue (Arg75; shown as cyan sticks) in the Vβ domain but it is away from the pMHC (approximately 22 Å) and CDRs. TCR α chain is light blue, β chain is blue, peptide is magenta, and MHC is green. Figure generated using PyMOL (www.pymol.org).
